# Dimensions of spiritual well-being in relation to physical and psychological symptoms: a cross-sectional study of advanced cancer patients admitted to a palliative care unit

**DOI:** 10.1186/s12904-023-01261-x

**Published:** 2023-09-14

**Authors:** Yilong Yang, Xinxin Zhao, Meng Cui, Yumei Wang

**Affiliations:** 1 Department of Health Policy and Management, School of Public Health, Hangzhou Normal University, Hangzhou, P.R. China; 2 Hospice Ward, Shengjing Hospital of China Medical University, No.39 Huaxiang Road, Tiexi District, Shenyang, 110022 P.R. China

**Keywords:** Spiritual well‐being, Palliative care, Advanced cancer, Cancer-related symptoms, Symptom management

## Abstract

**Objectives:**

Advanced cancer patients face various symptoms, which can cause physical and psychological distress. As a multidimensional construct, spiritual well-being (SWB) may be an inner resource for dealing with these problems. Our study explored the impact of different dimensions of SWB on physical and psychological symptoms in advanced cancer patients admitted to a palliative care unit.

**Methods:**

This cross-sectional study was conducted among 108 advanced cancer patients in the Hospice Ward, Shengjing Hospital of China Medical University. Patients completed questionnaires on SWB and cancer-related symptoms (insomnia, fatigue, pain, depression and anxiety) at the time of admission. Linear regression analysis was applied to determine the relationship between SWB (meaning, peace and faith) and symptom distress.

**Results:**

SWB accounted for an additional variance of cancer-related symptoms (17.8% to 44.4%). Meaning was negatively associated with insomnia (β = -0.516, *p* < 0.001) and fatigue (β = -0.563, *p* < 0.001). Peace and faith were related to lower psychological symptoms, while meaning represented a positive effect on anxiety (β = 0.275, *p* = 0.036). Higher peace was associated with lower cancer pain (β = -0.422, *p* < 0.001).

**Conclusions:**

Our findings suggested that achieving peace and faith appeared to function consistently as a positive resource for advanced cancer patients on depression, anxiety and pain, while meaning may serve to facilitate or hinder positive adjustment. Future studies should focus on the potential clinical implications by identifying the distinct dimension of SWB as symptom management targets in the palliative care practice.

**Supplementary Information:**

The online version contains supplementary material available at 10.1186/s12904-023-01261-x.

## Introduction

Advanced cancer patients suffer from avariety of physical and psychological symptoms associated with cancer diagnosis and treatment. Patients were often poly-symptomatic frequently experiencing cancer-related symptoms such as insomnia, fatigue, pain, depression, and anxiety [1, 2], which may contribute to substantial distress. Cancer-related symptoms tend to co-occur, and each of these symptoms can lead to the maintenance of the others. For instance, the positive relationship between pain, anxiety and preoperative insomnia was found in colorectal cancer patients [3], and poorly managed pain was associated with anxiety, sleep disturbance and fatigue in breast cancer survivors [4]. The pain-fatigue-depression symptom cluster was also present in prostate cancer survivors [5]. More importantly, patients with multiple symptoms were more likely to have multiplicative rather than additive distress [6]. Cancer-related symptoms can adversely influence functional activities and quality of life (QoL), decrease treatment compliance, and increase the risk for hospitalizations [1, 2, 7]. Thus, the interactions between insomnia, fatigue, pain, and depression/anxiety are complex, warranting interventions that focus not only on the routine symptom management, but also on the additional improvements in holistic cancer care.

Since spiritual well-being (SWB) is the fourth dimension of QoL in terminal patients, spiritual care has been considered as a fundamental component of palliative care (PC) [8, 9]. Spiritual care at the end of life has been proven to be important to PC patients [10]. There were notable relationships between spiritual domains and patient-centered PC outcomes [11]. However, despite its clinical significance, spiritual care often goes unrecognized and untreated in Chinese PC units. Given the immaturity of palliative medicine [12], addressing spiritual problems has not been a priority among health care professionals who carry out PC [13]. For advanced cancer patients receiving PC, their fundamental purpose is to control physical symptoms rather than to meet spiritual needs within short-term hospital stays [8, 12]. Therefore, a better understanding of the influence of SWB on cancer-related symptoms may contribute to an improved identification of spiritual care as one route to support symptom management.

SWB has been conceptualized as composed of three dimensions: meaning, peace, and faith [8, 14]. Meaning indicates the cognitive aspect of SWB and may contribute to the maintenance of stability and goals amid life challenges. Peace describes the affective domain of SWB and may be experienced when an individual has reached acceptance in the face of adversity. Faith is a sense of comfort or strength one derives from one’s spiritual beliefs as a foundation for understanding the world [8, 14]. SWB has been recognized by PC specialists as a positive coping resource to support adjustment to cancer-related symptoms [9–11]. Several studies demonstrated the positive influence of SWB on symptom distress, with supportive evidence that SWB was correlated with lower severity of pain, fatigue, sleep disturbance, and anxious/depressive symptoms [6–8]. Yet, professionals have not always found it easy to implement SWB within the holistic cancer care [15], which may be largely due to the multidimensional nature. To our knowledge, little is known about how the distinct dimensions of SWB jointly affect cancer-related symptoms. Examining only the composite score of SWB can lead PC specialists to overlook significant differences about the dimensions’ relations with symptom experience, which may result in that spiritual care remains the least developed and most neglected component of PC [10].

Meaning, peace and faith suggest different features of SWB, and they are not invariably linked with better adjustment to cancer-related experiences. A systematic review indicated that meaning and peace were related to better QoL, whereas faith was not consistently associated with health outcomes [16]. Increase in peace was related to lower depression and anxiety, while changes in meaning and faith were unrelated to psychological distress [17]. In longitudinal studies, meaning and peace were related to improved mental health and lower distress, and faith was associated with perceived growth [18]. Furthermore, the sense of faith may prompt feelings of negative emotions, such as when one experienced challenge that violated one’s views of spiritual beliefs [18]. Specially for QoL dimensions in cancer patients, meaning and peace were positively related to social/family and functional well-being, while faith had a negative effect on physical and emotional well-being [19]. The above findings lend support to the argument that the relationship of SWB dimensions with salutary outcomes may be inconsistent. Overall, despite the protective effect of SWB against maladjustment, there is limited knowledge about how meaning, peace and faith differentially influence multiple symptom experiences among advanced cancer patients in the PC unit.

Supporting SWB and its meaningful dimensions is an under-explored area which may yield future advancements in the simultaneous management of cancer-related symptoms. Moreover, there is a need to culturally reflect on differences in the dimensions of SWB [6]. The purpose of the current study was to evaluate whether specific dimensions of SWB functioned as a resource for Chinese adults experiencing cancer-related symptoms. Understanding the distinct dimension of SWB may help us address spiritual care incorporated into PC purposefully and thereby to support PC patients in dealing with physical and psychological symptoms.

## Methods

### Ethical approval statement

The study was approved by the Ethics committee on Human Experimentation of China Medical University (reference number: 720042321006479) and complied with the 1964 Helsinki Declaration and its later amendments. All participants provided informed written consent prior to participation.

### Study design and participants

A cross-sectional study was conducted of consecutive inpatients admitted to Shengjing Hospital of China Medical University between July 2019 and October 2020. Patients were recruited at the Hospice Ward as a baseline investigation of a prospective study [8]. Eligible patients were at least 18 years of age and had a confirmed diagnosis of solid cancer. Other eligibility criteria were that patients had a life expectancy < 6 months at the time of admission and provided clear responses to questionnaires in Chinese language. Exclusion criteria were that patients had (1) concomitant psychiatric disorders, (2) intellectual and/or cognitive impairments, (3) psychotropic drug usage within 7 days before admission, (4) drug or alcohol dependence, and (5) HIV-positive.

### Procedures

Eligible patients were identified through chart review and discussion with physicians. After explaining the study purpose, advanced cancer patients were asked to complete all the psychometric instruments within 24 h of admission to Hospice Ward. Demographic information and medical history were obtained through medical record review.

### Measures

#### Demographics and clinical variables

Demographic variables including age, gender, relationship status, and education were collected at admission. Diagnosis disclosure, first or subsequent visit, and Karnofsky Performance Scale (KPS) were determined by medical chart review.

#### SWB

The Functional Assessment of Chronic Illness Therapy-Spiritual Well-being (FACIT-Sp) consists of 12 items measuring SWB with three empirically validated subscales of 4 items: meaning, peace, and faith [20]. Participants rated on a five-point Likert-type scale (0 = “not at all” to 4 = “very much”). Subscale scores range from 0 to 16, with higher scores reflecting higher levels of SWB dimensions. Cronbach’s alphas for FACIT-Sp and its three subscales were 0.911, 0.76 (meaning), 0.703 (peace), and 0.914 (faith), respectively.

#### Insomnia

Insomnia was evaluated by the Athens Insomnia Scale (AIS) [21], which comprises 8 items rated on a 4-point Likert scale, ranging from 0 (no problem at all) to 3 (a very serious problem). The total score ranges from 0 to 24, with higher scores denoting higher insomnia. The Cronbach’s alpha for AIS in this study was 0.889.

#### Fatigue

Fatigue was assessed using the Cancer Fatigue Scale (CFS), which is a brief, valid, and feasible measure of fatigue for use with cancer patients [22]. The scale consists of 15 items, and each item is rated on a scale of 1 (not at all) to 5 (very much). Higher scores reveal more severe fatigue. The Cronbach’s alpha for the current sample was 0.866.

#### Pain

Cancer pain was assessed by the Numerical Rating Scales (NRS), which is one of the frequently used tools for assessing pain severity [23]. Patients were asked to verbally rate their pain on a scale from 0 to 10, with 0 equals no pain and 10 equals worst possible pain. The 11-point NRS is easy to administer verbally among advanced cancer patients admitted to the palliative setting [8, 14].

#### Depression and Anxiety

The Hospital Anxiety and Depression Scale (HADS) is a 14-item measure of depressive and anxious symptoms [24]. HADS consists of two subscales, with 7 items each rated on a 4-point Likert scale (0 = “not at all” to 3 = “very much indeed”). Higher scores on both subscales indicate more severe symptoms of depression and anxiety. Cronbach’s alphas for the depression and anxiety subscales were 0.877 and 0.865, respectively.

### Statistical analysis

Descriptive statistics of study variables were presented with median, mean, standard deviation (SD), 95% confidence intervals (CI), number (N), and percentage (%) as appropriate. The distributions of cancer-related symptoms in covariates were tested by independent sample* t* test and one-way analysis of variance (ANOVA). Pearson’s correlation was used to examine associations among the continuous variables. Hierarchical regression analysis was used to evaluate the association of SWB dimensions with symptom experience (i.e., insomnia, fatigue, pain, depression, and anxiety) after adjusting for covariates, which were related to outcomes in univariate analysis (*p* < 0.05). In Step 1, covariates were added. Due to the high correlations among the FACIT-Sp subscales, meaning, peace and faith were adjusted in the stepwise regression in Step 2. Data including R^2^, adjusted R^2^ (Adj. R^2^), R^2^-changes, F, standardization regression coefficients (β), and *p* value were provided for each step in the regression model. The Statistical Package for the Social Sciences (SPSS, version 18.0) was employed to perform the statistical analyses, with a two-tailed probability value of < 0.05 recognized to be statistically significant.

## Results

### Characteristics of patients

A convenience sample of 147 patients was originally recruited, and we received effective responses from 108 cancer patients (73.5%). The mean (SD) age was 65.23 ± 12.48 years. Approximately 75% of the patients were married or living with a partner, and 50.47% received middle school education. Most patients were aware of cancer diagnosis (65.71%), and were the first time to the Hospice Ward (77.78%). The mean (SD) of KPS was 47.83 ± 11.63, and 48 (45.28%) patients had a KPS score less than 50. Table 1 provided the descriptive statistics of SWB and cancer-related symptoms. The mean values were 24.12 ± 10.87 for SWB, 9.59 ± 3.74 for meaning, 7.67 ± 3.22 for peace, and 6.85 ± 4.98 for faith.

**Table 1:** Descriptive statistics for spiritual well-being and cancer-related symptoms

Variables	Mean	95% CI		SD	Median	Test range	Sample range
		Lower	Upper				
SWB	24.12	22.05	26.19	10.87	23.5	0-48	0-48
Meaning	9.59	8.88	10.31	3.74	10	0-16	0-16
Peace	7.67	7.06	8.29	3.22	7	0-16	0-16
Faith	6.85	5.91	7.8	4.98	6	0-16	0-16
Insomnia	12.59	11.51	13.67	5.66	12	0-24	2-24
Fatigue	53.56	51.81	55.31	9.17	53	15-75	28-74
Pain	6.41	5.8	7.02	3.18	7	0-10	0-10
Depression	10.55	9.51	11.6	5.48	10	0-21	0-21
Anxiety	10.01	9.08	10.94	4.88	9.5	0-21	0-21

*Abbreviation*: *SD* Standard deviation, *CL* Confidence interval, *SWB* Spiritual well-being

### Univariate analysis

As shown in Table 2, *t* test indicated the significant association of depression (*p* = 0.018) and anxiety (*p* = 0.038) with diagnosis disclosure. Advanced cancer patients who knew their cancer diagnosis reported higher scores of HADS subscales. The correlations between study variables were presented in Table 3. SWB and its dimensions were negatively associated with cancer-related symptoms (r = ranged from -0.373 to -0.687; *p* < 0.001). Additionally, meaning, peace and faith correlated highly with each other (*r* = ranged from 0.706 to 0.757; *p* < 0.001).

**Table 2:** Demographic and medical characteristics of patients and the distributions of cancer-related symptoms in categorical items

Variables	N(%)	Insomnia	*p*^a^	Fatigue	*p*^a^	Pain	*p*^a^	Depression	*p*^a^	Anxiety	*p*^a^
**Demographic information**											
Gender			0.977		0.906		0.939		0.897		0.743
Male	52 (48.15)	12.57±5.98		53.45±9.23		6.39±3.1		10.62±5.08		9.85±4.95	
Female	56 (51.85)	12.61±5.39		53.66±9.19		6.43±3.28		10.49±5.87		10.16±4.86	
Relationship status			0.873		0.396		0.983		0.418		0.206
Married, living with partner	80 (74.77)	12.47±5.42		53.01±8.48		6.4±3.15		10.8±5.33		10.31±4.83	
Living alone	27 (25.23)	12.67±6.34		55.02±11.13		6.42±3.38		9.8±6.04		8.93±4.99	
Education			0.544		0.741		0.845		0.441		0.614
Middle school	54 (50.47)	12.5±5.54		53.1±8.93		6.41±3.16		10.92±5.33		10.41±4.82	
High school	28 (26.17)	11.73±6.8		53.21±10.66		6.16±3.77		9.4±5.74		9.32±5.27	
Junior college or above	25 (23.36)	13.45±4.35		54.78±8.23		6.67±2.64		11.04±5.66		9.73±4.67	
**Medical information**											
Aware of cancer diagnosis			0.067		0.081		1		0.018		0.038
Yes	69 (65.71)	13.32±5.66		54.81±8.63		6.46±3.13		11.58±5.6		10.75±5.03	
No	36 (34.29)	11.19±5.47		51.5±9.97		6.46±3.32		8.93±4.87		8.67±4.36	
First time to PCU			0.836		0.394		0.884		0.76		0.732
Yes	84 (77.78)	12.53±5.76		53.16±9.1		6.43±3.27		10.64±5.41		10.1±4.89	
No	24 (22.22)	12.8±5.41		54.98±9.45		6.33±2.91		10.25±5.82		9.71±4.95	
KPS score (range 0-100)			0.098		0.423		0.415		0.482		0.721
≥ 50	58 (54.72)	11.73±5.56		52.8±9.34		6.15±3.31		10.19±5.3		9.83±5.12	
< 50	48 (45.28)	13.57±5.74		54.23±8.84		6.66±3.06		10.95±5.67		10.17±4.67	

*Abbreviation*: *KPS* Karnofsky Performance Scale, *PCU* Palliative care unit

^a^Independent sample *t* test and one-way analysis of variance were used

**Table 3:** Zero-order correlations (Pearson’s r) among continuous variables

Variables	1	2	3	4	5	6	7	8	9	10
1. Age	1	-0.013	-0.035	0.058	-0.039	-0.011	0.238*	0.048	-0.018	0.016
2. SWB		1	0.915***	0.88***	0.927***	-0.53***	-0.542***	-0.424***	-0.687***	-0.625***
3. Meaning			1	0.757***	0.756***	-0.513***	-0.57***	-0.375***	-0.619***	-0.481***
4. Peace				1	0.706***	-0.469***	-0.416***	-0.418***	-0.646***	-0.648***
5. Faith					1	-0.469***	-0.486***	-0.373***	-0.619***	-0.583***
6. Insomnia						1	0.523***	0.576***	0.575***	0.525***
7. Fatigue							1	0.457***	0.572***	0.487***
8. Pain								1	0.471***	0.501***
9. Depression									1	0.773***
10. Anxiety										1

*Abbreviation*: *SWB* Spiritual well-being

^*^p < 0.05 (two-tailed); ***p < 0.001 (two-tailed)

### Multivariate analysis

Tables 4 presented the hierarchical regression analysis of cancer-related symptoms after adjusting for covariates. The three dimensions of SWB accounted for an additional variance of cancer-related symptoms (17.8% to 44.4%) in Step 2. For insomnia-fatigue-pain symptoms, meaning or peace represented significant individual protective variables. Meaning was negatively associated with insomnia (β = -0.516, *p* < 0.001) and fatigue (β = -0.563, *p* < 0.001), and peace showed a significant association with cancer pain (β = -0.422, *p* < 0.001). Regarding psychological symptoms, peace and faith were negatively and significantly associated with depression and anxiety, but meaning represented a positive effect on anxiety (β = 0.275, *p* = 0.036). In addition, Additional file 1: Table A in the appendix also indicated the association between total score of FACIT-Sp and cancer-related symptoms, and SWB was proven to be a protective variable for these symptoms.

**Table 4:** Results of regression analyses exploring associations between dimensions of spiritual well-being and cancer-related symptoms

Symptom distress	Variables	Step 1			Step 2		
		β	t	*p*	β	t	*p*
Insomnia	Covariates						
	Age	-0.01	-0.107	0.915	-0.032	-0.375	0.708
	Gender	0.002	0.018	0.986	-0.029	-0.344	0.731
	SWB						
	Meaning				-0.516	-6.123	<0.001
	R^2^	0			0.265		
	Adj. R^2^	-0.019			0.244		
	R^2^-change	0			0.265		
	F	0.006			12.502		
	*p*	0.994			<0.001		
Fatigue	Covariates						
	Age	0.242	2.544	0.012	0.219	2.804	0.006
	Gender	0.036	0.383	0.703	0.003	0.036	0.971
	SWB						
	Meaning				-0.563	-7.228	<0.001
	R^2^	0.058			0.373		
	Adj. R^2^	0.04			0.355		
	R^2^-change	0.058			0.315		
	F	3.243			20.63		
	*p*	0.043			<0.001		
Pain	Covariates						
	Age	0.049	0.498	0.619	0.074	0.822	0.413
	Gender	0.012	0.127	0.899	0.016	0.175	0.862
	SWB						
	Peace				-0.422	-4.749	<0.001
	R^2^	0.002			0.18		
	Adj. R^2^	-0.017			0.157		
	R^2^-change	0.002			0.178		
	F	0.127			7.619		
	*p*	0.881			<0.001		
Depression	Covariates						
	Age	0.015	0.151	0.88	0.018	0.243	0.808
	Gender	-0.073	-0.743	0.459	-0.031	-0.425	0.671
	Aware of cancer diagnosis	0.244	2.456	0.016	0.155	2.067	0.041
	SWB						
	Peace				-0.419	-4.038	<0.001
	Faith				-0.293	-2.833	0.006
	R^2^	0.058			0.486		
	Adj. R^2^	0.03			0.46		
	R^2^-change	0.058			0.428		
	F	2.087			18.718		
	*p*	0.107			<0.001		
Anxiety	Covariates						
	Age	0.044	0.44	0.661	0.07	0.941	0.349
	Gender	-0.021	-0.213	0.831	0.031	0.412	0.681
	Aware of cancer diagnosis	0.215	2.145	0.034	0.15	1.98	0.05
	SWB						
	Peace				-0.61	-5.063	<0.001
	Faith				-0.333	-2.851	0.005
	Meaning				0.275	2.127	0.036
	R^2^	0.044			0.488		
	Adj. R^2^	0.015			0.457		
	R^2^-change	0.044			0.444		
	F	1.537			15.578		
	*p*	0.21			<0.001		

Three dimensions (meaning, peace, faith) were added in Step 2 adjusted by the stepwise regression due to the high correlations among the FACIT-Sp subscales

*Abbreviation*: *SWB* Spiritual well-being, *β* Standardized regression coefficient, *Adj.R*^*2*^ Adjusted R^2^

## Discussion

The current study is the first to examine the association between the distinct dimensions of SWB and symptom experience, as referred by advanced cancer patients receiving hospital-based PC. Our central finding was that different dimensions of SWB presented the distinct value in the adjustment to cancer-related symptoms independent of sample characteristics. The present study highlights the importance of spiritual care in the integrated PC by distinguishing the dimensions of SWB among advanced cancer patients at admission.

Several researchers demonstrated Meaning/Peace as one factor associated with physical and psychological consequences in cancer patients [6, 16, 18, 25]. However, the three-factor structure of FACIT-Sp reflected the conceptual difference between meaning (cognitive dimension) and peace (affective dimension) in terminal cancer patients [26], indicating that meaning and peace may differentially correlate with cancer-related symptoms. Our findings showed that a sense of meaning can be an inner resource for advanced cancer patients when facing insomnia and fatigue. One potential explanation may be the coping strategies that patients adopted in face of advanced cancer. Positive reframing coping style was a significant predictor of the Meaning dimension [27]. Patients found a new meaning and purpose in their existence by looking at cancer-related challenges from a distinct perspective or by trying to get the best out of adversity [27]. Thus, meaning as a cognitive component of SWB may alleviate insomnia and fatigue by enabling patients to reevaluate their own symptoms experience of the PC situation.

The positive meaning-anxiety link was contrary to previous studies that Meaning/Peace component had the significant buffering effect against psychological symptoms [6, 27, 28]. The possible explanation was that providing a cognitive reframing on patients’ declining health and imminent death may be not beneficial for dealing with anxiety in response to cancer-related stress. Considered as one of emotional issues, anxious symptoms may be exacerbated from a cognitive perspective when patients strived for the meaning or purpose in their lives. However, the explanation about the negative effects of meaning is rather exploratory, and the association between meaning and anxiety deserves further investigations.

Concerning the affective domain, peace may be the dimension of SWB most closely associated with psychological symptoms. Under standardized estimate (β) sequence, peace had the strongest buffering effect against depression and anxiety, in agreement with previous studies found that there was a significant negative association between peace and psychological distress in cancer patients [17, 29, 30]. Bovero et al. demonstrated that a sense of peace contributed to relieving symptoms of depression and anxiety by adopting emotional regulation strategies (e.g., self-distraction and behavioral disengagement) [27]. According to positive emotions in positive psychology, peace and harmony are related to engaging in meaning‐in‐life behaviors [17, 31], which may reflect mechanisms underlying the adaptive value of finding peace as an affective support for advanced cancer patients.

Peace also emerged as the only dimension of SWB that was related to pain severity in our study. Cancer pain is a complex subjective experience associated with bio-psycho-spiritual factors [8, 14]. Depression and anxiety were associated with more severe pain, resulting in that cancer pain became intractable in the presence of psychological distress [32]. Sleight et al. found that meaning/peace buffered the negative effect of anxiety on physical well-being in cancer survivors. [25] We speculated that a sense of peace that can reduce depression and anxiety may further relieve pain severity, which may be a mechanism underlying the peace-pain association.

A greater faith was associated with lower psychological symptoms, which was in contrast to previous findings that meaning/peace had a greater association with improved mental health and lower psychological distress than faith in cancer patients [16–18]. Particularly, faith hindered the positive adjustment to cancer, such as an association between faith and adverse health outcomes [6, 19]. The mixed results when evaluating faith’s role in cancer patients may reflect cultural differences. Faith involves perceived comfort and strength derived from spiritual beliefs and practices. Faith of cancer patients who adhere to religion can be achieved by an omnipotent power or through a religious practice [7, 27]. Nevertheless, reliance on faith when one experiences events that violate one’s connection to a higher power may lead to negative religious beliefs (e.g., feeling abandoned by God and spiritual discontent), and thus may hinder favorable outcomes compared with meaning and peace [18, 29]. In opposition to a religious society, Chinese people tend to discover the meaning in life, find self-worth in the world, and explore the essence of university [19]. The search of faith may be a spiritual resource in helping Chinese cancer patients foster positive adjustment to psychological symptoms by repositioning themselves properly in the universe and establishing harmonious association with nature [19]. Therefore, a sense of faith may provide emotional regulation and greater resilience while facing depression and anxiety, making symptoms more tolerable and controllable.

### Clinical implications

Our findings highlighted the distinct adaptive value of meaning, peace and faith on cancer-related symptoms, with potential clinical implications for improving symptom management. Although several interventions which are important in spiritual process have been shown to increase spirituality [8, 17], meaning, peace and faith have not been specifically distinguished and targeted in advanced cancer patients. Health care providers should explore the relevance of the distinct dimensions of SWB in the PC practice to soften symptom distress. For instance, pain is the most common and distressing symptom in advanced cancer, resulting in the particularly high priority of pain management in PC [14, 33]. Besides the pharmacological strategies, future researches should assess whether increasing peace as an intervention target in the plan of PC can decrease pain severity.

### Limitations

Our findings should be interpreted within the context of study limitations. First, the study design was cross-sectional, without demonstrating a causal relationship between SWB and cancer-related symptoms. Next, the recruitment of patients was a convenience sampling in one university hospital, which limited the generalizability of our findings. Additionally, spiritual distress was not evaluated in our study while it may have clinical implications. Spiritual distress is an area worthy of future study in relation to physical and psychological symptoms [7]. Finally, our findings should be further replicated in larger studies and with chronic diseases other than cancer.

## Conclusions

The present study suggested that the distinct dimensions of SWB empowered individuals to transcend cancer-related symptoms in a PC unit. Specifically, achieving peace and faith appeared to function consistently as a positive resource for advanced cancer patients on depression, anxiety and pain, while meaning may serve to facilitate (insomnia and fatigue) or hinder (anxiety) positive adjustment. Our findings have implications for incorporating spiritual care in the holistic PC and identifying the most important dimension of SWB as symptom management targets.

### Supplementary Information

**Additional file 1:** **Table A.** Results of regression analyses exploring associations between spiritual well-being and cancer-related symptoms.

**Additional file 2.** Raw data.

## Data Availability

All data generated or analyzed during this study are included in this published article [and its supplementary information files].

## Abbreviations

PC: Palliative care
SWB: Spiritual well-being
QoL: Quality of life
NRS: Numerical Rating Scales
KPS: Karnofsky Performance Scale
FACIT-Sp: Functional Assessment of Chronic Illness Therapy-Spiritual Well-being
AIS: Athens Insomnia Scale
CFS: Cancer Fatigue Scale
NRS: Numerical Rating Scales
HADS: Hospital Anxiety and Depression Scale
SD: Standard deviation
CI: Confidence intervals
ANOVA: One-way analysis of variance
