# Supplementary material for: Dimensions of spiritual well-being in relation to physical and psychological symptoms: a cross-sectional study of advanced cancer patients admitted to a palliative care unit
Source: BMC Palliat Care. 2023 Sep 14;22:137. doi: 10.1186/s12904-023-01261-x (PMC10500771; doi:10.1186/s12904-023-01261-x)
Supplement: Supplementary file 1 — Additional file 1: Table A. Results of regression analyses exploring associations between spiritual well-being and cancer-related symptoms. [file 12904_2023_1261_MOESM1_ESM.docx]

**Table A.** Results of regression analyses exploring associations between spiritual well-being and cancer-related symptoms

| Symptom distress | Variables | Step 1 | | |  | Step 2 | | |
| --- | --- | --- | --- | --- | --- | --- | --- | --- |
|  |  | β | t | *p* |  | β | t | *p* |
| Insomnia | Covariates |  |  |  |  |  |  |  |
|  | Age | -0.01 | -0.107 | 0.915 |  | -0.017 | -0.375 | 0.839 |
|  | Gender | 0.002 | 0.018 | 0.986 |  | 0.003 | -0.344 | 0.968 |
|  | SWB |  |  |  |  | -0.53 | -6.381 | <0.001 |
|  | R^2^ | 0 |  |  |  | 0.281 |  |  |
|  | Adj. R^2^ | -0.019 |  |  |  | 0.261 |  |  |
|  | R^2^-change | 0 |  |  |  | 0.281 |  |  |
|  | F | 0.006 |  |  |  | 13.576 |  |  |
|  | *p* | 0.994 |  |  |  | <0.001 |  |  |
| Fatigue | Covariates |  |  |  |  |  |  |  |
|  | Age | 0.242 | 2.544 | 0.012 |  | 0.236 | 2.96 | 0.004 |
|  | Gender | 0.036 | 0.383 | 0.703 |  | 0.038 | 0.479 | 0.633 |
|  | SWB |  |  |  |  | -0.539 | -6.812 | <0.001 |
|  | R^2^ | 0.058 |  |  |  | 0.349 |  |  |
|  | Adj. R^2^ | 0.04 |  |  |  | 0.33 |  |  |
|  | R^2^-change | 0.058 |  |  |  | 0.291 |  |  |
|  | F | 3.243 |  |  |  | 18.564 |  |  |
|  | *p* | 0.043 |  |  |  | <0.001 |  |  |
| Pain | Covariates |  |  |  |  |  |  |  |
|  | Age | 0.049 | 0.498 | 0.619 |  | 0.044 | 0.489 | 0.626 |
|  | Gender | 0.012 | 0.127 | 0.899 |  | 0.014 | 0.154 | 0.878 |
|  | SWB |  |  |  |  | -0.423 | -4.767 | <0.001 |
|  | R^2^ | 0.002 |  |  |  | 0.181 |  |  |
|  | Adj. R^2^ | -0.017 |  |  |  | 0.158 |  |  |
|  | R^2^-change | 0.002 |  |  |  | 0.179 |  |  |
|  | F | 0.127 |  |  |  | 7.678 |  |  |
|  | *p* | 0.881 |  |  |  | <0.001 |  |  |
| Depression | Covariates |  |  |  |  |  |  |  |
|  | Age | 0.015 | 0.151 | 0.88 |  | -0.005 | -0.068 | 0.946 |
|  | Gender | -0.073 | -0.743 | 0.459 |  | -0.038 | -0.518 | 0.605 |
|  | Aware of cancer diagnosis | 0.244 | 2.456 | 0.016 |  | 0.139 | 1.85 | 0.067 |
|  | SWB |  |  |  |  | -0.657 | -8.996 | <0.001 |
|  | R^2^ | 0.058 |  |  |  | 0.48 |  |  |
|  | Adj. R^2^ | 0.03 |  |  |  | 0.459 |  |  |
|  | R^2^-change | 0.058 |  |  |  | 0.421 |  |  |
|  | F | 2.087 |  |  |  | 23.038 |  |  |
|  | *p* | 0.107 |  |  |  | <0.001 |  |  |
| Anxiety | Covariates |  |  |  |  |  |  |  |
|  | Age | 0.044 | 0.44 | 0.661 |  | 0.026 | 0.321 | 0.749 |
|  | Gender | -0.021 | -0.213 | 0.831 |  | 0.011 | 0.136 | 0.892 |
|  | Aware of cancer diagnosis | 0.215 | 2.145 | 0.034 |  | 0.119 | 1.467 | 0.146 |
|  | SWB |  |  |  |  | -0.601 | -7.64 | <0.001 |
|  | R^2^ | 0.044 |  |  |  | 0.396 |  |  |
|  | Adj. R^2^ | 0.015 |  |  |  | 0.372 |  |  |
|  | R^2^-change | 0.044 |  |  |  | 0.352 |  |  |
|  | F | 1.537 |  |  |  | 16.398 |  |  |
|  | *p* | 0.21 |  |  |  | <0.001 |  |  |

Abbreviation: SWB, spiritual well-being; β, standardized regression coefficient; Adj.R^2^, adjusted R^2^.
